# Supplementary material for: Estimation of the prevalence of substance use by wastewater-based epidemiology study in four cities of Guangdong, China
Source: PLoS One. 2025 Apr 9;20(4):e0320141. doi: 10.1371/journal.pone.0320141 (PMC11981132; doi:10.1371/journal.pone.0320141)
Supplement: S1 Table — (DOCX) [file pone.0320141.s001.docx]

**S1 Table. Information on WWTPs in Four Cities in Guangdong Province**

| Serial Number | Sampling city | Sewage number | The population served (10000 people) | Daily inflow rate (cubic meters/day) |
| --- | --- | --- | --- | --- |
| 1 | Guangzhou | GZ-1 | 25 | 30000 |
| 2 |  | GZ-2 | 41 | 150000 |
| 3 |  | GZ-3 | 16 | 15000 |
| 4 |  | GZ-4 | 5 | 6000 |
| 5 |  | GZ-5 | 5 | 48000 |
| 6 |  | GZ-6 | 13 | 60000 |
| 7 |  | GZ-7 | 5.8 | 50000 |
| 8 |  | GZ-8 | 7.2 | 20000 |
| 9 |  | GZ-9 | 40 | 50000 |
| 10 |  | GZ-10 | 25 | 75000 |
| 11 |  | GZ-11 | 68 | 400000 |
| 12 |  | GZ-12 | 25.5 | 20000 |
| 13 |  | GZ-13 | 15 | 80000 |
| 14 |  | GZ-14 | 40 | 97000 |
| 15 |  | GZ-15 | 0.75 | 10000 |
| 16 |  | GZ-16 | 80.6 | 235000 |
| 17 |  | GZ-17 | 13 | 100000 |
| 18 |  | GZ-18 | 60 | 250000 |
| 19 |  | GZ-19 | 66.87 | 153000 |
| 20 |  | GZ-20 | 111.12 | 230900 |
| 21 |  | GZ-21 | 23 | 40000 |
| 22 |  | GZ-22 | 150 | 550000 |
| 23 |  | GZ-23 | 76.25 | 500000 |
| 24 |  | GZ-24 | 303.6 | 1200000 |
| 25 |  | GZ-25 | 174.83 | 650000 |
| 26 | Shantou | ST-1 | 95 | 265632 |
| 27 |  | ST-2 | 3 | 7608 |
| 28 |  | ST-3 | 28 | 108000 |
| 29 |  | ST-4 | 42.5 | 79992 |
| 30 |  | ST-5 | 31.5 | 86208 |
| 31 |  | ST-6 | 20 | 49992 |
| 32 |  | ST-7 | 21.38 | 42000 |
| 33 |  | ST-8 | 17.5 | 13992 |
| 34 |  | ST-9 | 3.3 | 15000 |
| 35 |  | ST-10 | 5.96 | 13584 |
| 36 |  | ST-11 | 7.17 | 9600 |
| 37 |  | ST-12 | 9 | 27168 |
| 38 |  | ST-13 | 13.5 | 14688 |
| 39 |  | ST-14 | 3.65 | 37368 |
| 40 |  | ST-15 | 26.35 | 42312 |
| 41 |  | ST-16 | 16 | 18144 |
| 42 |  | ST-17 | 13 | 22056 |
| 43 |  | ST-18 | 13 | 9648 |
| 44 |  | ST-19 | 43.5 | 44904 |
| 45 |  | ST-20 | 28.5 | 34512 |
| 46 |  | ST-21 | 29.87 | 78816 |
| 47 |  | ST-22 | 4.2 | 12000 |
| 48 | Qingyuan | QY-1 | 15.4 | 80000 |
| 49 |  | QY-2 | 9.6 | 40000 |
| 50 |  | QY-3 | 4.4 | 20000 |
| 51 |  | QY-4 | 8 | 80000 |
| 52 |  | QY-5 | 7.9 | 40000 |
| 53 |  | QY-6 | 11.5 | 30000 |
| 54 |  | QY-7 | 10 | 40000 |
| 55 |  | QY-8 | 12 | 32892 |
| 56 |  | QY-9 | 13 | 20000 |
| 57 |  | QY-10 | 12 | 20000 |
| 58 |  | QY-11 | 20 | 50567 |
| 59 |  | QY-12 | 18 | 34652 |
| 60 |  | QY-13 | 5 | 12000 |
| 61 |  | QY-14 | 3 | 9000 |
| 62 | Maoming | MM-1 | 26.3 | 25000 |
| 63 |  | MM-2 | 28.92 | 30000 |
| 64 |  | MM-3 | 48 | 94479 |
| 65 |  | MM-4 | 15 | 39400 |
| 66 |  | MM-5 | 26 | 46000 |
| 67 |  | MM-6 | 30 | 47449 |
| 68 |  | MM-7 | 4 | 63 |
