# Supplementary material for: Estimation of the prevalence of substance use by wastewater-based epidemiology study in four cities of Guangdong, China
Source: PLoS One. 2025 Apr 9;20(4):e0320141. doi: 10.1371/journal.pone.0320141 (PMC11981132; doi:10.1371/journal.pone.0320141)
Supplement: S2 Table — (DOCX) [file pone.0320141.s002.docx]

**S2 Table. Concentration values of target drugs detected in all WWTPs**

| Sample number | Morphine (ng/L) | 6-Aetylmorphine(ng/L) | Amphetamine (ng/L) | Methamphetamine (ng/L) | Ketamine (ng/L) | Norketamine (ng/L) | MDMA（ng/L) | MDA  （ng/L) | Cocaine (ng/L) | Benzoylecgonine（ng/L) |
| --- | --- | --- | --- | --- | --- | --- | --- | --- | --- | --- |
| GZ-1 | 27.80 | 1.47 | 4.08 | 89.08 | 27.19 | 0.41 | <LOD | <LOD | <LOD | <LOD |
| GZ-2 | 4.27 | <LOD | 7.72 | 172.95 | 5.76 | 0.44 | <LOD | <LOD | <LOD | <LOD |
| GZ-3 | 8.50 | 0.39 | 1.07 | 24.79 | 24.76 | <LOD | <LOD | <LOD | <LOD | <LOD |
| GZ-4 | 15.32 | 0.86 | 6.47 | 153.59 | 1.27 | <LOD | <LOD | <LOD | <LOD | <LOD |
| GZ-5 | 6.79 | 0.80 | 1.09 | 71.55 | 25.31 | <LOD | <LOD | <LOD | <LOD | <LOD |
| GZ-6 | <LOD | <LOD | 3.89 | 133.53 | 4.77 | <LOD | <LOD | <LOD | <LOD | <LOD |
| GZ-7 | 1.70 | 0.30 | 4.18 | 77.55 | 7.56 | 0.42 | <LOD | <LOD | <LOD | <LOD |
| GZ-8 | 35.92 | 1.29 | 6.14 | 151.08 | 8.43 | <LOD | <LOD | <LOD | <LOD | <LOD |
| GZ-9 | 2.71 | <LOD | 9.97 | 208.21 | <LOD | <LOD | <LOD | <LOD | <LOD | <LOD |
| GZ-10 | 7.76 | <LOD | 2.68 | 26.1 | 23.14 | <LOD | <LOD | <LOD | <LOD | <LOD |
| GZ-11 | 7.51 | 0.30 | 16.09 | 251.72 | 8.76 | <LOD | <LOD | <LOD | <LOD | <LOD |
| GZ-12 | <LOD | <LOD | 13.49 | 238.56 | 9.88 | <LOD | <LOD | <LOD | <LOD | <LOD |
| GZ-13 | 14.79 | 0.32 | <LOD | <LOD | 8.65 | <LOD | <LOD | <LOD | <LOD | <LOD |
| GZ-14 | 28.38 | 3.56 | 5.85 | 65.89 | <LOD | <LOD | <LOD | <LOD | <LOD | <LOD |
| GZ-15 | 18.01 | 1.70 | 4.69 | 165.23 | 3.83 | <LOD | <LOD | <LOD | <LOD | <LOD |
| GZ-16 | 6.86 | <LOD | 1.17 | 12.66 | 3.09 | <LOD | <LOD | <LOD | <LOD | <LOD |
| GZ-17 | 9.00 | 0.31 | 7.50 | 200.02 | 15.3 | <LOD | <LOD | <LOD | <LOD | <LOD |
| GZ-18 | <LOD | <LOD | 5.6 | 125.04 | <LOD | <LOD | <LOD | <LOD | <LOD | <LOD |
| GZ-19 | 20.17 | 0.45 | 1.98 | 29.26 | 5.90 | 0.42 | <LOD | <LOD | <LOD | <LOD |
| GZ-20 | 25.45 | 2.74 | 6.74 | 123.62 | 10.13 | 0.41 | <LOD | <LOD | <LOD | <LOD |
| GZ-21 | 2.82 | <LOD | 1.31 | 17.37 | 21.79 | <LOD | <LOD | <LOD | <LOD | <LOD |
| GZ-22 | 4.90 | 1.41 | 2.04 | 22.42 | 7.28 | 0.50 | <LOD | <LOD | <LOD | <LOD |
| GZ-23 | 2.64 | 0.30 | 2.47 | 35.97 | 16.42 | <LOD | <LOD | <LOD | <LOD | <LOD |
| GZ-24 | 1.63 | <LOD | 2.35 | 30.30 | 1.33 | <LOD | <LOD | <LOD | <LOD | <LOD |
| ST-1 | 10.25 | <LOD | 0.72 | 51.28 | 38.08 | 2.80 | <LOD | <LOD | <LOD | <LOD |
| ST-2 | 11.55 | <LOD | 3.03 | 80.24 | 49.88 | 3.05 | <LOD | <LOD | <LOD | <LOD |
| ST-3 | 34.69 | 3.06 | 7.73 | 103.06 | 15.59 | <LOD | <LOD | <LOD | <LOD | <LOD |
| ST-4 | 9.35 | <LOD | 1.22 | 55.94 | 9.70 | <LOD | <LOD | <LOD | <LOD | <LOD |
| ST-5 | 12.39 | <LOD | 2.51 | 88.00 | 20.43 | 1.40 | <LOD | <LOD | <LOD | <LOD |
| ST-6 | 20.33 | 0.87 | 1.41 | 55.27 | 24.554 | 1.31 | <LOD | <LOD | <LOD | <LOD |
| ST-7 | 24.77 | 1.51 | 3.15 | 86.65 | 35.62 | 2.28 | <LOD | <LOD | <LOD | <LOD |
| ST-8 | 15.34 | 0.74 | 8.09 | 97.43 | 28.14 | 1.64 | <LOD | <LOD | <LOD | <LOD |
| ST-9 | 32.44 | 2.69 | <LOD | <LOD | <LOD | <LOD | <LOD | <LOD | <LOD | <LOD |
| ST-10 | 16.87 | 0.79 | 9.65 | 109.88 | 22.84 | 0.88 | <LOD | <LOD | <LOD | <LOD |
| ST-11 | 21.81 | 1.27 | 8.50 | 102.18 | 17.73 | <LOD | <LOD | <LOD | <LOD | <LOD |
| ST-12 | 21.01 | 1.04 | 2.17 | 65.54 | 10.13 | <LOD | <LOD | <LOD | <LOD | <LOD |
| ST-13 | 54.27 | 4.05 | 7.75 | 98.26 | 11.96 | <LOD | <LOD | <LOD | <LOD | <LOD |
| ST-14 | 15.30 | <LOD | 6.16 | 97.26 | 25.33 | 1.80 | <LOD | <LOD | <LOD | <LOD |
| ST-15 | 18.75 | 0.92 | 1.36 | 79.90 | 31.16 | 2.24 | <LOD | <LOD | <LOD | <LOD |
| ST-16 | 8.99 | <LOD | 12.68 | 134.46 | 20.10 | 0.76 | <LOD | <LOD | <LOD | <LOD |
| ST-17 | 5.27 | <LOD | 2.25 | 70.26 | 41.02 | 3.00 | <LOD | <LOD | <LOD | <LOD |
| ST-18 | 29.56 | 2.36 | 6.81 | 90.19 | 11.58 | <LOD | <LOD | <LOD | <LOD | <LOD |
| ST-19 | 2.70 | <LOD | 9.10 | 109.54 | 29.92 | 2.06 | <LOD | <LOD | <LOD | <LOD |
| ST-20 | 5.25 | <LOD | 1.46 | 63.80 | 18.28 | <LOD | <LOD | <LOD | <LOD | <LOD |
| ST-21 | 1.75 | <LOD | 2.35 | 89.30 | 13.78 | <LOD | <LOD | <LOD | <LOD | <LOD |
| ST-22 | <LOD | <LOD | 0.70 | 48.05 | 3.68 | <LOD | <LOD | <LOD | <LOD | <LOD |
| QY-1 | 18.81 | 0.30 | 2.53 | 57.87 | 8.11 | <LOD | <LOD | <LOD | <LOD | <LOD |
| QY-2 | 10.09 | <LOD | 8.10 | 148.2 | 33.27 | 0.50 | 0.31 | <LOD | <LOD | <LOD |
| QY-3 | 26.51 | 0.59 | 8.75 | 147.39 | 28.94 | 0.30 | <LOD | <LOD | <LOD | <LOD |
| QY-4 | 9.23 | <LOD | 0.31 | 1.36 | 5.59 | <LOD | <LOD | <LOD | <LOD | <LOD |
| QY-5 | 5.40 | <LOD | <LOD | <LOD | 2.45 | <LOD | <LOD | <LOD | <LOD | <LOD |
| QY-6 | 29.68 | 2.28 | 3.24 | 131.12 | 27.71 | <LOD | <LOD | <LOD | <LOD | <LOD |
| QY-7 | 1.59 | <LOD | 7.78 | 141.28 | 28.53 | 0.31 | <LOD | <LOD | <LOD | <LOD |
| QY-8 | 28.1 | 1.88 | 8.04 | 141.58 | 26.81 | <LOD | <LOD | <LOD | <LOD | <LOD |
| QY-9 | 25.54 | 1.01 | 10.72 | 270.58 | 32.00 | 0.32 | 0.39 | <LOD | <LOD | <LOD |
| QY-10 | 4.56 | <LOD | 2.03 | 25.4 | 5.11 | <LOD | <LOD | <LOD | <LOD | <LOD |
| QY-11 | 4.08 | <LOD | 6.55 | 133.91 | 28.59 | 0.31 | <LOD | <LOD | <LOD | <LOD |
| QY-12 | <LOD | <LOD | 1.31 | 18.29 | 4.08 | <LOD | <LOD | <LOD | <LOD | <LOD |
| QY-13 | 34.63 | 2.75 | 2.29 | 32.24 | 6.00 | <LOD | <LOD | <LOD | <LOD | <LOD |
| QY-14 | 8.54 | <LOD | 2.63 | 68.52 | <LOD | <LOD | <LOD | <LOD | <LOD | <LOD |
| MM-1 | 11.52 | 0.79 | 5.67 | 137.98 | 5.20 | 0.38 | <LOD | <LOD | <LOD | 0.36 |
| MM-2 | 16.5 | 1.38 | 4.05 | 66.37 | 5.05 | 0.37 | <LOD | <LOD | <LOD | <LOD |
| MM-3 | 3.42 | <LOD | 1.44 | 57.31 | 4.66 | 0.33 | <LOD | <LOD | <LOD | <LOD |
| MM-4 | 7.12 | 0.59 | 1.98 | 65.98 | 2.26 | 0.33 | <LOD | <LOD | <LOD | 0.31 |
| MM-5 | 7.12 | 0.30 | 3.52 | 66.08 | 1.49 | <LOD | <LOD | <LOD | <LOD | <LOD |
| MM-6 | 3.09 | <LOD | 0.71 | 4.52 | <LOD | <LOD | <LOD | <LOD | <LOD | <LOD |
| MM-7 | 1.07 | <LOD | <LOD | <LOD | 0.99 | <LOD | <LOD | <LOD | <LOD | <LOD |
