# Supplementary material for: Estimation of the prevalence of substance use by wastewater-based epidemiology study in four cities of Guangdong, China
Source: PLoS One. 2025 Apr 9;20(4):e0320141. doi: 10.1371/journal.pone.0320141 (PMC11981132; doi:10.1371/journal.pone.0320141)
Supplement: S3 Table — (DOCX) [file pone.0320141.s003.docx]

**S3 Table.** Mean influent loads (mg/1000 inh/d) of drug residues in four cities of Guangdong, 2023 to 2024

| Drug Residues | Guangzhou | | Qingyuan | | Shantou | | Maoming | |
| --- | --- | --- | --- | --- | --- | --- | --- | --- |
|  | 2023 | 2024 | 2023 | 2024 | 2023 | 2024 | 2023 | 2024 |
| Morphine | 9±6 ^a^ | 8±5 ^a^ | 16±10 ^a^ | 12±7 ^a^ | 7±5 ^a^ | 11±7 ^a^ | 2±1^a^ | 2±1 ^a^ |
| 6-AMA | 1±1 ^a^ | 1±1 ^a^ | 2±1 ^a^ | 1±1 ^a^ | 1±1 ^a^ | 2±2 ^a^ | 0.2±0.1 ^a^ | 0.1±0.1 ^a^ |
| Amphetamine | 2±1 ^a^ | 3±2 ^a^ | 2±1 ^a^ | 5±3 ^a^ | 6±4 ^a^ | 7±5 ^a^ | 1±1^a^ | 3±3 ^a^ |
| Methamphetamine | 94±83 ^a^ | 110±90 ^a^ | 76±66 ^a^ | 88±72 ^a^ | 38±27 ^a^ | 45±31 ^a^ | 24±17^a^ | 52±26 ^a^ |
| Ketamine | 1±1 | 4±2 | 2±1 | 4±3 | 2±1 | 5±3 | 0.5±0.3 | 1±1 |
| Norketamine | 0.02±0.01 ^a^ | 0.08±0.05 ^ab^ | 0.03±0.01 ^a^ | 0.09±0.06 ^ab^ | 0.5±0.2 ^a^ | 0.7±0.5 ^a^ | 0.01±0.01^a^ | 0.03±0.02 ^a^ |
| MDMA | - | - | 0.02±0.01 | - | - | - | - | - |
| MDA | - | - | - | - | - | - | - | - |
| Benzoylecgonine | - | - | - | - | - | - | 0.03±0.02 | 0.05±0.04 |
| Cocaine | - | - | - | - | - | - | - | - |

Note: All values are mean±SD of influent loads； a means followed by different superscript alphabets in each line are significantly different (P≤0.05) among different cities. b Means followed by different superscript alphabets in each line are significantly different (P< 0.05) among different years.
